# Common and distinct variation in data fusion of designed experimental data

**DOI:** 10.1007/s11306-019-1622-2

**Published:** 2019-12-03

**Authors:** Masoumeh Alinaghi, Hanne Christine Bertram, Anders Brunse, Age K. Smilde, Johan A. Westerhuis

**Affiliations:** 1 Department of Food Science, Aarhus University, Aarslev, Denmark; 2 Comparative Pediatrics and Nutrition, Department of Veterinary and Animal Sciences, Faculty of Health and Medical Sciences, University of Copenhagen, Copenhagen, Denmark; 3 Biosystems Data Analysis, Swammerdam Institute for Life Sciences, University of Amsterdam, Amsterdam, The Netherlands

**Keywords:** ANOVA-simultaneous component analysis (ASCA), Multiset data analysis, Data integration, NMR metabolomics, Concave penalty

## Abstract

**Introduction:**

Integrative analysis of multiple data sets can provide complementary information about the studied biological system. However, data fusion of multiple biological data sets can be complicated as data sets might contain different sources of variation due to underlying experimental factors. Therefore, taking the experimental design of data sets into account could be of importance in data fusion concept.

**Objectives:**

In the present work, we aim to incorporate the experimental design information in the integrative analysis of multiple designed data sets.

**Methods:**

Here we describe penalized exponential ANOVA simultaneous component analysis (PE-ASCA), a new method for integrative analysis of data sets from multiple compartments or analytical platforms with the same underlying experimental design.

**Results:**

Using two simulated cases, the result of simultaneous component analysis (SCA), penalized exponential simultaneous component analysis (P-ESCA) and ANOVA-simultaneous component analysis (ASCA) are compared with the proposed method. Furthermore, real metabolomics data obtained from NMR analysis of two different brains tissues (hypothalamus and midbrain) from the same piglets with an underlying experimental design is investigated by PE-ASCA.

**Conclusions:**

This method provides an improved understanding of the common and distinct variation in response to different experimental factors.

**Electronic supplementary material:**

The online version of this article (10.1007/s11306-019-1622-2) contains supplementary material, which is available to authorized users.

## Introduction

With an increased availability of data from multiple sources, data fusion is one of the main challenges in omics sciences, such as metabolomics (Crockford et al. 2006; Smilde et al. 2005b). Different pieces of information can be captured from the same set of objects with the same or with different features. Collected data from multiple compartments (i.e. biological fluids and tissues) (Noguchi et al. 2006) or analytical platforms (Crockford et al. 2006) with the same set of objects are examples of this situation. Integrative analysis of these multiple data sets with a shared mode (i.e. object or variable direction) can be useful for improving the understanding of the studied biological system. A number of different methods are proposed to capture the same latent phenomena in different data sets (i.e. common variation) as well as the phenomena which can only be found in one data matrix (i.e. distinct variation) (Smilde et al. 2017; Van Deun et al. 2012, 2013). The common variation can be used to explain association between different data sets such as same pattern of variability, while the distinct variation can provide a better understanding of specific source of variability in each data set. Methods such as generalized SVD (GSVD) (Alter et al. 2003), joint and individual variation explained (JIVE) (Lock et al. 2013), distinct and common simultaneous component analysis (DISCO-SCA) (Schouteden et al. 2013), two block orthogonal partial least squares (O2-PLS) (Trygg and Wold 2003) attempt to capture both common and distinct sources of variation across different data sets. However, these methods use different approaches in selecting the number of common and distinct components, and in many cases do not agree (Måge et al. 2019; van der Kloet et al. 2016) . Recently, methods such as structural learning and integrative decomposition (SLIDE) (Gaynanova and Li 2017) and penalized exponential simultaneous component analysis (P-ESCA) (Song et al. 2019) with the possibility of modeling partially shared variation and component selection (i.e. identification of the number of components for common and distinct variation) are developed. In the P-ESCA model, a group sparsity constraint has been used to find the global and local common and distinct components using a concave penalty.

Metabolomics data sets are highly complex as they mostly contain different underlying designed experimental factors, such as treatment, time, diets, doses of the drug, etc. (Antti et al. 2004). Even though data fusion is crucial for understanding the relationship between different data sets, current data fusion methods (Alter et al. 2003; Gaynanova and Li 2017; Lock et al. 2013; Schouteden et al. 2013; Song et al. 2019; Trygg and Wold 2003) do not take the experimental complexity of the data sets into account. This means that different sources of variation explaining the contribution of the experimental design are not described in the data fusion models, which could hamper an easy interpretation of the common and distinct sources of variation. ANOVA simultaneous component analysis (ASCA) (Jansen et al. 2005b; Smilde et al. 2005a) is widely used for the analysis of the complicated multivariate data with many measured variables from a designed metabolomics study. ASCA can decompose the data to low-rank submatrices that can be assigned to different experimental factors and their interactions.

In this paper, we aim to merge ASCA with fusion methods to provide the common and distinct sources of variation related to each factor, interaction or their combination as well as the individual differences of the objects that cannot be explained by designed factors. This can be crucial for understanding the underlying information in multiple biological data sets and allows for an easy interpretation of the common and distinct latent components for separated variation induced by different designed factors. The proposed penalized exponential ANOVA simultaneous component analysis (PE-ASCA) model presented here can deal with the complexity of the designed biological data in data fusion concept. Therefore, two simulated data sets and a real nuclear magnetic resonance (NMR) metabolomics data set with experimental design are analyzed by the PE-ASCA model. Significance of the experimental factors will be quantified by calculating the p values using a permutation test (Vis et al. 2007; Zwanenburg et al. 2011). The metabolomics data, analyzed in this paper, is part of an intervention study (Alinaghi et al. 2019; Møller et al. 2011) where data from multiple compartments are available for the same piglets to investigate bloodstream infection. However, the experimental design of the newborn piglets cause the need for a fusion model which can deal with the complexity of the designed biological data. Neonatal sepsis, the clinical manifestation of a severe bloodstream infection in early life, may injure the developing brain and cause neurodevelopmental impairment (Stoll et al. 2004). Efforts to reduce systemic inflammation by supplementation of the bovine colostrum (COL) might reduce the risk of brain injury and improve the long-term outcomes. Colostrum, the first milk after birth in mammals (Møller et al. 2011), contains a multitude of proteins and peptides with anti-inflammatory and anti-microbial properties which seem to protect the immature newborns from harmful inflammatory reactions and brain injury. Therefore, high-resolution magic-angle spinning (HR-MAS) NMR spectroscopy was applied to study how neonatal bloodstream infection would diet-dependently change the metabolome of different brain tissues.

## Theory

In this section we will shortly introduce the SCA, ASCA and P-ESCA models and discuss their properties. SCA is a general extension of PCA when multiple data sets of the same samples are available, ASCA is a method for decomposing multivariate data into effect matrices, according to an experimental design, after which the effect matrices can be explored separately. The P-ESCA model is able to find common and distinctive variation in multiple sets of data. Then, using the same notation we will introduce the PE-ASCA model and discuss its superior properties over the previous models when there is an experimental design underlying two or more data sets measured on the same samples. Properties of these models are summarized in Table 1.

**Table 1:** Properties of the SCA, ASCA, P-ESCA and PE-ASCA methods

Methods	Experimentally designed data		Decomposition into factor effect matrices	Decomposition into Common and distinctive variation	Decomposition into factor effect matrices within common and distinctive variation
	NO	YES			
SCA	X	X			
ASCA		X	X		
P-ESCA	X	X		X	
PE-ASCA		X	X	X	X

### SCA model

Simultaneous component analysis (SCA) (Jansen et al. 2005a; Ten Berge et al. 1992) can simultaneously analyzed multiple data sets with a shared object mode (measuring different features for the same set of objects). Suppose ${\mathbf{X}}_{n}$ contains the same number of rows *I* and possibly different number of columns $J_{n},$ then SCA can decompose each data set according to Eq. (1).

1 $${\mathbf{X}}_{n} = {\mathbf{TP}}_{n}^{T} + \varvec{ }{\mathbf{E}}_{n} \varvec{ }$$

where ${\mathbf{X}}_{n} \in \varvec{ }{\text{R}}^{{I \times J_{n} }},$ where $n = 1, \ldots ,N$ corresponds to the various multivariate data sets, whose rows contain the measurement of objects $i = 1, \ldots ,I$ and columns contain the measurement of variables $j_{n} = 1, \ldots ,J_{n}.$ ${\mathbf{T}} \in \varvec{ }{\text{R}}^{I \times R}$ is the common score matrix with $r = 1, \ldots ,R$ number of significant common components and ${\mathbf{P}}_{n} \in \varvec{ }{\text{R}}^{{J_{n} \times R}}$ contains the relevant loadings for each data ${\mathbf{X}}_{n}.$ ${\mathbf{E}}_{n} \in \varvec{ }{\text{R}}^{{I \times J_{n} }}$ is the residual matrix.

### ASCA model

Analysis of variance (ANOVA) (Searle 1971) can be used to determine the effect of different experimental factors on a single measured response. Based on the basic idea of the ANOVA, ASCA can decompose a multivariate data set to orthogonal and independent sub-matrices explaining the variation induced by experimental factors. For the situation with more than one data matrix with a shared object mode, the ASCA decomposition can be applied for each data set ${\mathbf{X}}_{n}$ separately. The ASCA decomposition for multivariate data with a two-way experimental design of factors *α* and *β* containing different levels can be seen in Eq. (2).

2 $${\mathbf{X}}_{n} = 1{\mathbf{m}}_{n}^{\text{T}} + \varvec{ }{\mathbf{X}}_{n\alpha } + \varvec{ }{\mathbf{X}}_{n\beta } + \varvec{ }{\mathbf{X}}_{n\alpha \beta } + \varvec{ }{\mathbf{X}}_{n,ind} \varvec{ }$$

where **1** is a column vector of ones of size *I*, ${\mathbf{m}}_{n}$ is a column vector of size $J_{n}$ with overall means of responses per variable; matrices ${\mathbf{X}}_{n\alpha },$ ${\mathbf{X}}_{n\beta }$ and ${\mathbf{X}}_{n\alpha \beta } \varvec{ } \in \varvec{ }{\text{R}}^{{I \times J_{\text{n}} }} \varvec{ }$ contain the estimated values of the experimental factor *α*, *β* and the interaction *αβ*, while each row of the matrices ${\mathbf{X}}_{n\alpha }$ and ${\mathbf{X}}_{n\beta }$ is related to one level of the factors *α* and *β* respectively. ${\mathbf{X}}_{n,ind} \in \varvec{ }{\text{R}}^{{I \times J_{n} }} \varvec{ }$ is a matrix containing all the individual differences between the objects after subtracting all effect matrices. As all rows within the same level of a factor have the same values, the ASCA decomposed-matrices are low-rank matrices. SCA is used to diminish the number of variables $J_{n}$ to a lower number of latent components in matrices ${\mathbf{X}}_{n\alpha },$ ${\mathbf{X}}_{n\beta }$ and ${\mathbf{X}}_{n\alpha \beta } \varvec{ }$ (Eq. (3)).

3 $${\mathbf{X}}_{nl} = {\mathbf{T}}_{nl} {\mathbf{P}}_{nl}^{\text{T}} + \varvec{ }{\mathbf{E}}_{nl} \varvec{ } l \in \left\{ \alpha \right.,\beta ,\left. {\alpha \beta ,ind} \right\}$$

where ${\mathbf{T}}_{nl} \in \varvec{ }{\text{R}}^{{I \times R_{\text{l}} }}$ is the SCA score matrix of ASCA effect for factor *l* with $R_{l}$ number of significant components and ${\mathbf{P}}_{nl} \in \varvec{ }{\text{R}}^{{J_{n} \times R_{\text{l}} }}$ contains the relevant loadings. To identify the ASCA model, each loading matrix has orthonormal columns, hence, ${\mathbf{P}}_{nl}^{\text{T}} {\mathbf{P}}_{nl} = {\mathbf{I}},l \in \left\{ \alpha \right.,\beta ,\left. {\alpha \beta ,ind} \right\}.$ $\varvec{E}_{nl} \in \varvec{ }{\text{R}}^{{I \times J_{n} }}$ contains the residual part of ${\mathbf{X}}_{nl}$ that is not described by the SCA model. To prevent the overestimation of the experimental effects, a permutation test can be performed on the effect matrices. The null hypothesis H_0_ then represents there is no experimental effect larger than estimated effects from permuted data between the level averages in the score matrices **T**_*l*_ (Zwanenburg et al. 2011).

**Fig. 1:**
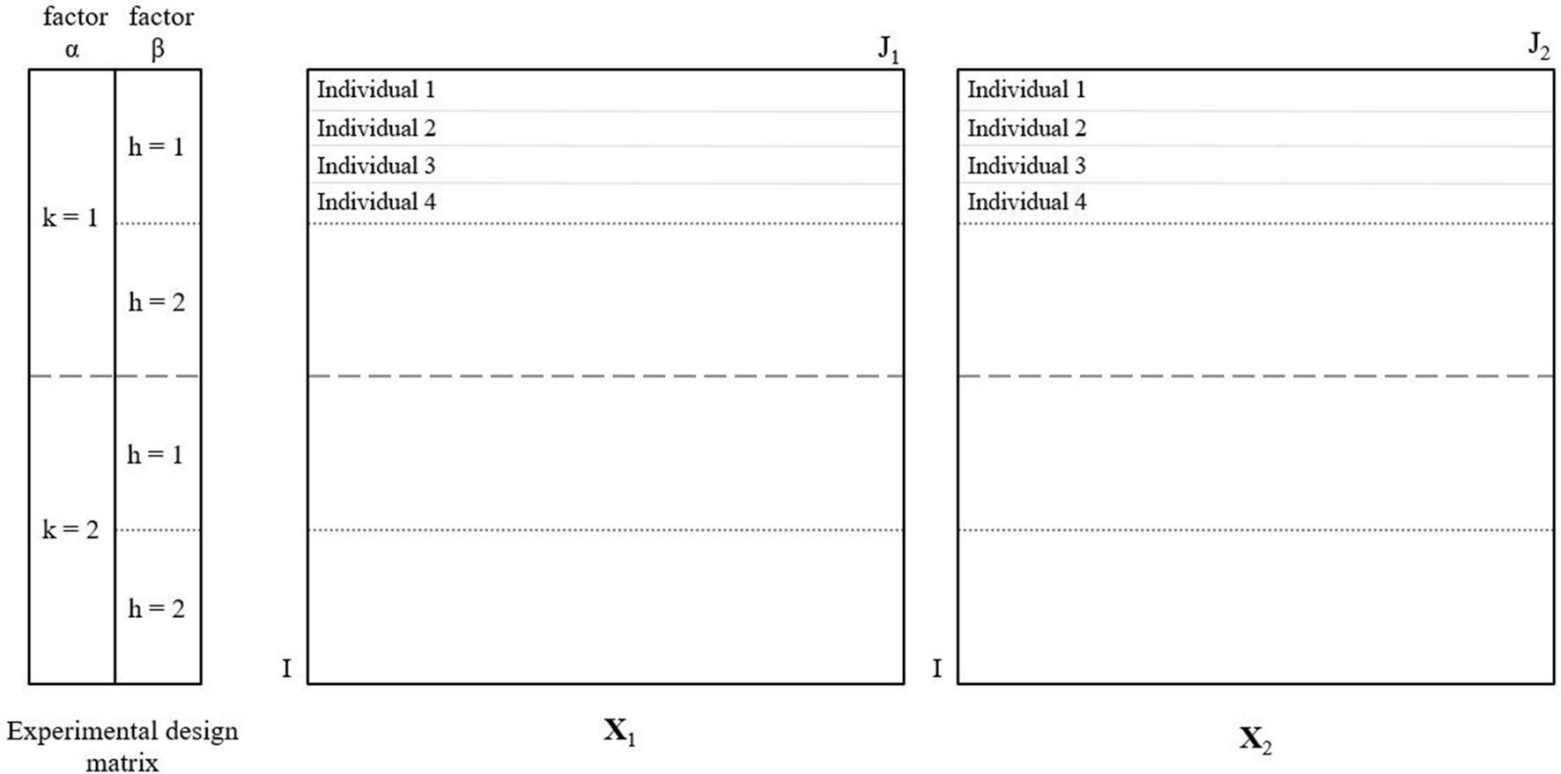
Structure of the data sets for integrative analysis. The left matrix represents the experimental design matrix with the first column containing the levels of *k* = 1, …, *K* (*K* = 2) of factor *α* and second column containing the levels of *h* = 1, …, *H* (*H* = 2) of factor *β*. Multiple data sets, ${\mathbf{X}}_{1}$ and ${\mathbf{X}}_{2},$ contain the same number of rows *I* and possibly a different number of columns $J_{1}$ and $J_{2},$ respectively. Each individual can be represented as a specific level of factor *α* and *β*

For the situations that factor *β* is not independently of interest, its combination with the interaction $({\mathbf{X}}_{n\beta } + \varvec{ }{\mathbf{X}}_{n\alpha \beta }$) can be evaluated, especially when the time factor is desired (Jansen et al. 2005b). In this paper, the combination *β* + *αβ* is considered for investigation. Combination of the time and treatment effect is interpreted as the trend of changes in treatment levels over time. If we assume that factor *α* has *K* levels $k = 1, \ldots ,K$ and factor *β* has *H* levels $h = 1, \ldots ,H,$ each individual can be represented as a combination of specific level of factor *α* and *β*; i.e. $i_{kh} = 1, \ldots ,I_{kh}$ (Fig. 1). The rank of the matrix describing factor *α* is $\left( {K - 1} \right)$ and the rank for the factor matrix related to *β* is $\left( {H - 1} \right).$ The rank of the combination of factor β and interaction is $\left( {K - 1} \right)H.$

### P-ESCA model

According to the P-ESCA model (Song et al. 2019), common and distinct components can be disentangled from multiple data sets by applying structured sparsity on the SCA loading matrix. Fused data matrices can be decomposed to low dimensional matrices of common and distinct variation according to Eq. (4).

4 $${\mathbf{X}}_{n} = {\mathbf{X}}_{nc} + \varvec{ }{\mathbf{X}}_{nd} + \varvec{ }{\mathbf{E}}_{n} = \varvec{ }{\mathbf{T}}_{c} {\mathbf{P}}_{nc}^{T} + {\mathbf{T}}_{nd} {\mathbf{P}}_{nd}^{T} + \varvec{ }{\mathbf{E}}_{n},$$

where subscript *c* stands for the common variation and subscript *d* stands for the distinct variation. For the simplicity, local common variation is not considered here and ${\mathbf{X}}_{nc}$ contains the global common variation. ${\mathbf{E}}_{n}$ contains the nonsystematic variation. For the integrative analysis of multiple data sets with shared objects, the P-ESCA disentangles a score matrix with the size of *I* × *R* where $r = 1, \ldots ,R$ represents the number of significant components (for common and distinct combined) and a loading of size $(J_{1} + \ldots + J_{n} ) \times R$ with a sparsity pattern. Assuming the integrative analysis of two data sets $$\left[ {\begin{array}{*{20}c} {{\mathbf{X}}_{1} } & {{\mathbf{X}}_{2} } \\ \end{array} } \right] = {\mathbf{TP}}^{\text{T}}$$ where ${\mathbf{T}}^{\text{T}} {\mathbf{T}} = {\mathbf{I}},$ then the structure of the loading $\left\{ {{\mathbf{P}}_{n} } \right\}_{n = 1}^{2} \in \varvec{ }{\text{R}}^{{J_{n} \times R}}$ is given by in Eq. (5).

5 $${\mathbf{P}} = \left( {\begin{array}{*{20}c} {{\mathbf{P}}_{1} } \\ {{\mathbf{P}}_{2} } \\ \end{array} } \right) = \left( {\begin{array}{*{20}c} {{\mathbf{P}}_{1,1} } & {{\mathbf{P}}_{1,2} } & 0 \\ {{\mathbf{P}}_{2,1} } & 0 & {{\mathbf{P}}_{2,3} } \\ \end{array} } \right)$$

where ${\mathbf{P}}_{1,1}$ and ${\mathbf{P}}_{2,1}$ represent loadings of the common variation for ${\mathbf{X}}_{1}$ and ${\mathbf{X}}_{2},$ respectively. ${\mathbf{P}}_{1,2}$ and ${\mathbf{P}}_{2,3}$ represent loadings of the distinct variation of ${\mathbf{X}}_{1}$ and ${\mathbf{X}}_{2},$ respectively. By incorporating Eq. (5) in the P-ESCA model presented in Eq. (4), the following decomposition can be obtained for the integrative analysis of two data sets (Eq. (6)).

6 $$\begin{aligned} {\mathbf{X}}_{1} & = {\mathbf{T}}_{c} {\mathbf{P}}_{1,1} + {\mathbf{T}}_{1d} {\mathbf{P}}_{1,2} + \varvec{ }{\mathbf{E}}_{1} \\ {\mathbf{X}}_{2} & = {\mathbf{T}}_{c} {\mathbf{P}}_{2,1} + {\mathbf{T}}_{2d} {\mathbf{P}}_{2,3} + \varvec{ }{\mathbf{E}}_{2} \\ \end{aligned}$$

In cases with the integrative analysis of more than two data sets, there might be local common components, which can also be disentangled with the P-ESCA model. However, this is not of interest in this example since we only have two blocks of data.

### Data fusion of multiple designed experimental data sets

For incorporating the experimental design in the data fusion, two approaches are feasible: (i) first disentangle the common and distinct latent components of the data sets by P-ESCA and then, decompose common and distinct variation into the matrices explaining different experimental design factors by ASCA, or (ii) first decompose each data set into the matrices explaining different experimental design factors by ASCA and then, disentangle the common and distinct latent components of each factor matrices by P-ESCA. In the noiseless case both approaches lead to the same solution and further discussion on the comparison of these two approaches can be found in the supplementary material (Eqs. (S1)–(S5) and Fig. S1). P-ESCA estimates a noise level from the data to find the structured loadings. As ASCA already removes the individual variation from the data, P-ESCA has more difficulties finding the correct common and distinct structure after ASCA decomposition. Therefore, the first approach has been considered for further analysis in this paper.

The ASCA model can be incorporated on the P-ESCA decomposition to disentangle the common and distinct latent components for separated variation induced by different design factors (Eq. (7)).

7 $${\mathbf{X}}_{n} = 1{\mathbf{m}}_{n}^{\text{T}} + \varvec{ }{\mathbf{X}}_{nc\alpha } + \varvec{ }{\mathbf{X}}_{{nc\left( {\beta + \alpha \beta } \right)}} + {\mathbf{X}}_{nd\alpha } + {\mathbf{X}}_{{nd\left( {\beta + \alpha \beta } \right)}} + \varvec{ }{\mathbf{X}}_{nc,ind} + \varvec{ }{\mathbf{X}}_{nd,ind} + \varvec{ }{\mathbf{E}}_{n} \varvec{ }$$

Therefore, decomposition of multiple data sets is according to Eq. (8), which represents the PE-ASCA model.

8 $${\mathbf{X}}_{n} = \varvec{ }1{\mathbf{m}}_{n}^{\text{T}} + {\mathbf{T}}_{c\alpha } {\mathbf{P}}_{nc\alpha }^{\text{T}} + \varvec{ }{\mathbf{T}}_{{c\left( {\beta + \alpha \beta } \right)}} {\mathbf{P}}_{{nc\left( {\beta + \alpha \beta } \right)}}^{\text{T}} + {\mathbf{T}}_{c,ind} {\mathbf{P}}_{nc,ind}^{\text{T}} + {\mathbf{T}}_{nd\alpha } {\mathbf{P}}_{nd\alpha }^{\text{T}} + {\mathbf{T}}_{{nd\left( {\beta + \alpha \beta } \right)}} {\mathbf{P}}_{{nd\left( {\beta + \alpha \beta } \right)}}^{\text{T}} + {\mathbf{T}}_{nd,ind} {\mathbf{P}}_{nd,ind}^{\text{T}} + \varvec{ }{\mathbf{E}}_{n} \varvec{ }$$

where ${\mathbf{T}}_{c\alpha } \in \varvec{ }{\text{R}}^{{I \times R_{{{\text{c}}\upalpha }} }}$ is the matrix containing the common components explaining the contribution of factor *α* on the basis of ${\mathbf{P}}_{nc\alpha } \in \varvec{ }{\text{R}}^{{J_{n} \times R_{c\alpha } }}.$ ${\mathbf{T}}_{{c\left( {\beta + \alpha \beta } \right)}} \in \varvec{ }{\text{R}}^{{I \times R_{{c\left( {\beta + \alpha \beta } \right)}} }}$ is the matrix containing common components explaining the combined contribution of factor *β* and interaction *αβ* on the basis of ${\mathbf{P}}_{{nc\left( {\beta + \alpha \beta } \right)}} \in \varvec{ }{\text{R}}^{{J_{n} \times R_{{nc\left( {\beta + \alpha \beta } \right)}} }}.$ ${\mathbf{T}}_{c,ind} \in \varvec{ }{\text{R}}^{{I \times R_{c,ind} }}$ is the matrix containing the common components explaining the contribution of individual differences on the basis of ${\mathbf{P}}_{nc,ind} \in \varvec{ }{\text{R}}^{{J_{n} \times R_{c,ind} }}.$ ${\mathbf{T}}_{nd\alpha } \in \varvec{ }{\text{R}}^{{I \times R_{nd\alpha } }}$ is the matrix containing distinct components explaining the contribution of the factor *α* on the basis of ${\mathbf{P}}_{nd\alpha } \in \varvec{ }{\text{R}}^{{J_{n} \times R_{nd\alpha } }}.$ ${\mathbf{T}}_{{nd\left( {\beta + \alpha \beta } \right)}} \in \varvec{ }{\text{R}}^{{I \times R_{{nd\left( {\beta + \alpha \beta } \right)}} }}$ is the matrix containing distinct components explaining the contribution of factor *β* and interaction *αβ* on the basis of ${\mathbf{P}}_{{nd\left( {\beta + \alpha \beta } \right)}} \in \varvec{ }{\text{R}}^{{J_{n} \times R_{{nd\left( {\beta + \alpha \beta } \right)}} }}.$ ${\mathbf{T}}_{nd,ind} \in \varvec{ }{\text{R}}^{{I \times R_{nd,ind} }}$ is the matrix containing distinct components explaining the contribution of the individual differences on the basis of ${\mathbf{P}}_{nd, ind} \in \varvec{ }{\text{R}}^{{J_{n} \times R_{nd,ind} }}.$

The loadings of the model for integrative analysis of two data sets (*n*=2) is according to Eq. (9).

9 $$\left( {\begin{array}{*{20}c} {{\mathbf{P}}_{1} } \\ {{\mathbf{P}}_{2} } \\ \end{array} } \right) = \left( {\begin{array}{*{20}c} {\begin{array}{*{20}c} {{\mathbf{P}}_{1,1\alpha } } & {{\mathbf{P}}_{{1,2\left( {\beta + \alpha \beta } \right)}} } & {{\mathbf{P}}_{1,3,ind} } \\ {{\mathbf{P}}_{2,1\alpha } } & {{\mathbf{P}}_{{2,2\left( {\beta + \alpha \beta } \right)}} } & {{\mathbf{P}}_{2,3,ind} } \\ \end{array} } & {\begin{array}{*{20}c} {{\mathbf{P}}_{1,4\alpha } } & 0 & {{\mathbf{P}}_{{1,6\left( {\beta + \alpha \beta } \right)}} } \\ 0 & {{\mathbf{P}}_{2,5\alpha } } & 0 \\ \end{array} } & {\begin{array}{*{20}c} 0 & {{\mathbf{P}}_{1,8,ind} } & 0 \\ {{\mathbf{P}}_{{2,7\left( {\beta + \alpha \beta } \right)}} } & 0 & {{\mathbf{P}}_{2,9,ind} } \\ \end{array} } \\ \end{array} } \right)$$

where the first, second and third columns represent the common variation contributing to *α*, *β* + *αβ* effects, and individual differences, respectively. The fourth and fifth columns represent the distinct sources of variation contributing to factor *α* in first and second data sets, respectively. The sixth and seventh columns represent distinct sources of variation contributing to *β* + *αβ* effect in first and second data sets, respectively. The eighth and ninth columns represent the distinct sources of variation contributing to the individual variation in first and second data sets, respectively.

In the data sets with missing values, the problem can be handled by a missing value based cross validation for model selection. More explanation on the cross validation procedure can be found elsewhere (Song et al. 2019). In case of an unbalanced design, where the number of subject per group is not the same, an ad hoc resampling approach can be used, as was done in this paper, or one could use generalized linear models as was suggested by Thiel et al. (Thiel et al. 2017). Properties of the SCA, ASCA, P-ESCA and PE-ASCA methods can be found in Table 1.

## Results

### Simulated data

**Fig. 2:**
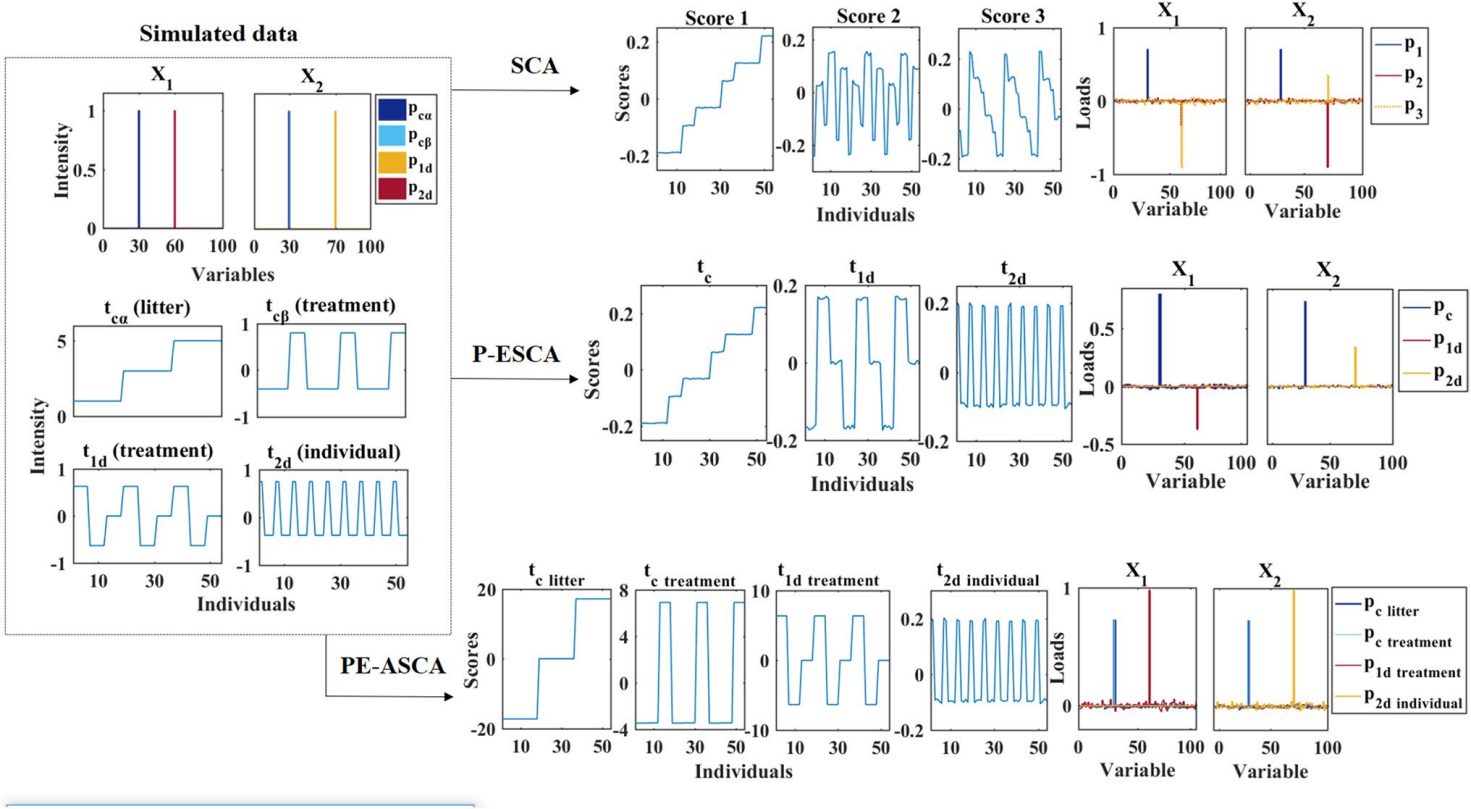
Simulated data for case A and the scores and loadings obtained by SCA, P-ESCA and PE-ASCA model

In this section, we investigate the performance of the PE-ASCA for identification of the common and distinct components contributed to each designed factor. Accordingly, the simulated data sets will be studied to compare the results of the proposed method with SCA and P-ESCA (case A), as well as ASCA (case B). In each case, two data sets are simulated for the integrative analysis (i.e. *n* = 2). The design matrix for simulated data sets is as follows (Eq. (10)).

10 $${\text{design matrix}} = \left[ {\begin{array}{*{20}c} {\begin{array}{*{20}c} 1 & 1 \\ 1 & 2 \\ 1 & 3 \\ \end{array} } \\ {\begin{array}{*{20}c} \vdots & \vdots \\ \end{array} } \\ {\begin{array}{*{20}c} 2 & 1 \\ 2 & 2 \\ 2 & 3 \\ \end{array} } \\ {\begin{array}{*{20}c} \vdots & \vdots \\ \end{array} } \\ {\begin{array}{*{20}c} 3 & 1 \\ 3 & 2 \\ 3 & 3 \\ \end{array} } \\ {\begin{array}{*{20}c} \vdots & \vdots \\ \end{array} } \\ \end{array} } \right]$$

where the first column represents different levels of factor *α* and the second column represents the levels of factor *β*. To keep the model simple, only one common component is considered for each designed factor, i.e. one common factor *α* and one common factor *β*. This simulation exemplifies the situation in which data sets ${\mathbf{X}}_{1}$ and ${\mathbf{X}}_{2}$ are obtained by analytical measurement of two different biological compartments of the same piglets (six biological replicates measured over 100 variables in both cases). The spectral loadings are simulated in a way that resemble a simplified metabolomics data set (Fig. 2).

Two cases are simulated to illustrate the advantages of the PE-ASCA from different aspects. In case A, the results of the PE-ASCA are compared with the integrative analysis with SCA and P-ESCA which are not considering the experimental design of the data. In case B, the PE-ASCA is compared with the ASCA modeling of different data sets without considering the integrative analysis. Matlab (version R2016b, MathWorks Inc., United States) was used for data analysis and visualization.

#### Case A

In this case, the simulated data sets are generated according to Eq. (11).

11 $${\mathbf{X}}_{1} = \varvec{ }{\mathbf{t}}_{c\alpha } {\mathbf{p}}_{1c\alpha }^{T} + {\mathbf{t}}_{c\beta } {\mathbf{p}}_{1c\beta }^{T} + {\mathbf{t}}_{1d\beta } {\mathbf{p}}_{1d\beta }^{T} + \varvec{ }{\mathbf{E}}_{1}$$

$${\mathbf{X}}_{2} = \varvec{ }{\mathbf{t}}_{c\alpha } {\mathbf{p}}_{2c\alpha }^{T} + {\mathbf{t}}_{c\beta } {\mathbf{p}}_{2c\beta }^{T} + {\mathbf{t}}_{2d,ind} {\mathbf{p}}_{2d,ind}^{T} + \varvec{ }{\mathbf{E}}_{2}$$

where ${\mathbf{t}}_{c\alpha }$ is the common component contributed to the variation of factor *α* (litter effect); ${\mathbf{t}}_{c\beta }$ is the common component contributed to the variation of *β* (treatment effect);${\mathbf{t}}_{1d\beta }$ is the distinct component of first data contributed to the variation of *β* (treatment effect); ${\mathbf{t}}_{2d,ind}$ is the distinct component of second data contributed to the individual variation. The simulated score matrices are independent and orthogonal to each other. The error matrices ${\mathbf{E}}_{1}$ and ${\mathbf{E}}_{2}$ are from a normal distribution with an average of zero and a standard deviation of 0.01. Signal-to-noise ratios for both ${\mathbf{X}}_{1}$ and ${\mathbf{X}}_{2}$ are 35.

In this case, no interaction is considered between litter and treatment factors. The litter effect, with three levels (i.e. level 1, 2 and 3), is explaining differences in piglets born from different sows. The treatment effect has three levels where level one relates to infected piglets, level two represents untreated infected piglets treated with supplementation, and level three represents the uninfected control piglets (CON). The simulated common and distinct components can be seen in the left part of Fig. 2. The common litter component (${\mathbf{t}}_{c\alpha }$) is simulated with an increasing effect from level one to level three. The infected group is separated from the other two groups as a common treatment component (${\mathbf{t}}_{c\beta }$). A distinct component explaining the treatment effect is present in the first data set (${\mathbf{t}}_{1d}$), where separation of all three treatment groups can be seen in this component. The distinct component of the second data set (${\mathbf{t}}_{2d,ind}$) is explaining the individual variation of piglets and is not explaining any contribution to the designed factors. Variable 30 is responsible for both common litter and treatment variation, while variable 60 is responsible for distinct treatment variation of first data and variable 70 is responsible for individual variation in second data (Fig. 2).

To evaluate the performance of the model in capturing the correct common and distinct components of designed factors, all resolved profiles of the PE-ASCA are visualized and compared with the result of SCA and P-ESCA (Fig. 2). The first score of the SCA shows the overall litter and common treatment effects which are corresponding to variable 30. The second and third scores are explaining the distinct variation in both data sets, while the second score is mostly contributing to the individual variation in the second data and the third score is mostly explaining the distinct treatment factor in first data. Generally, SCA could not resolve the simulated factors correctly. The integrative analysis of two data sets by P-ESCA model resulted in one common component explaining 54.3% variation of the first data and 54.1% variation of the second data. This common component contains information of both litter and treatment effect. On the other hand, the P-ESCA model is able to disentangle the distinct effects, while ${\mathbf{p}}_{1d}$ is only explaining variable 60 in first data and ${\mathbf{p}}_{2d}$ is contributing to variable 70 in the second data. The distinct component ${\mathbf{t}}_{1d},$ which represents the separation of all three treatment groups, explains 16.1% of the variation of the first data. The distinct component ${\mathbf{t}}_{2d}$ is explaining 16.2% of the variation in the second data, which represents the individual variation in ${\mathbf{X}}_{2}.$ Even though the results of P-ESCA show that the model is capable of separating the common and distinct factors, ${\mathbf{t}}_{c}$ is capturing the combination of both common litter and treatment effects.

In the PE-ASCA model, the common variation is represented by two common components describing the experimental effects *α* and *β*. The results show the correct separation of all simulated factors by considering the experimental design of data. Therefore, ASCA on common variation of ${\mathbf{X}}_{1}$ and ${\mathbf{X}}_{2}$ resulted in the correct estimation of common litter and common treatment effect as separate components (${\mathbf{t}}_{c\; litter}$ and ${\mathbf{t}}_{c\; treatment}$ respectively). A permutation test, performed on the effect matrices, showed that the litter and treatment effects are both significant with a p value = 0.001 for 1000 iterations. Performing the ASCA on distinct variation of the first data resulted in ${\mathbf{t}}_{d\; treatment}$ which is contributed to the variation of the treatment factor specific to the first data with a significant p value = 0.001 for 1000 iterations. ASCA on the distinct variation of the second data could not explain any significant effect to the experimental factors, however an individual pattern of the variation can be revealed in the second data. The corresponding loadings separately show the correct variables for the common and distinct variation.

#### Case B

In this case, the simulated data sets are generated according to Eq. (12).

12 $$\begin{aligned} {\mathbf{X}}_{1} & = {\mathbf{t}}_{c\alpha } {\mathbf{p}}_{1c\alpha }^{T} + {\mathbf{t}}_{{c\left( {\beta + \alpha \beta } \right)}} {\mathbf{p}}_{{1c\left( {\beta + \alpha \beta } \right)}}^{T} + {\mathbf{t}}_{{1d\left( {\beta + \alpha \beta } \right)}} {\mathbf{p}}_{{1d\left( {\beta + \alpha \beta } \right)}}^{T} + {\mathbf{E}}_{1} \\ {\mathbf{X}}_{2} & = {\mathbf{t}}_{c\alpha } {\mathbf{p}}_{2c\alpha }^{T} + {\mathbf{t}}_{{c\left( {\beta + \alpha \beta } \right)}} {\mathbf{p}}_{{2c\left( {\beta + \alpha \beta } \right)}}^{T} + {\mathbf{E}}_{2} \\ \end{aligned}$$

$${\mathbf{X}}_{2} = {\mathbf{t}}_{c\alpha } {\mathbf{p}}_{2c\alpha }^{T} + {\mathbf{t}}_{{c\left( {\beta + \alpha \beta } \right)}} {\mathbf{p}}_{{2c\left( {\beta + \alpha \beta } \right)}}^{T} + {\mathbf{E}}_{2}$$

where ${\mathbf{t}}_{c\alpha }$ is the common component contributed to the variation of factor *α* (time effect); ${\mathbf{t}}_{{c\left( {\beta + \alpha \beta } \right)}}$ is the common component contributed to the variation of *β* + *αβ* (combination of treatment and treatment × time effect);${\mathbf{t}}_{{1d\left( {\beta + \alpha \beta } \right)}}$ is the distinct component of first data set contributed to the variation of *β* + *αβ* (combination of treatment and treatment × time effect). The error matrices ${\mathbf{E}}_{1}$ and ${\mathbf{E}}_{2}$ are from a standard normal distribution with an average of zero and a standard deviation of 0.01. Signal-to-noise ratios for ${\mathbf{X}}_{1}$ and ${\mathbf{X}}_{2}$ are 38 and 33, respectively.

**Fig. 3:**
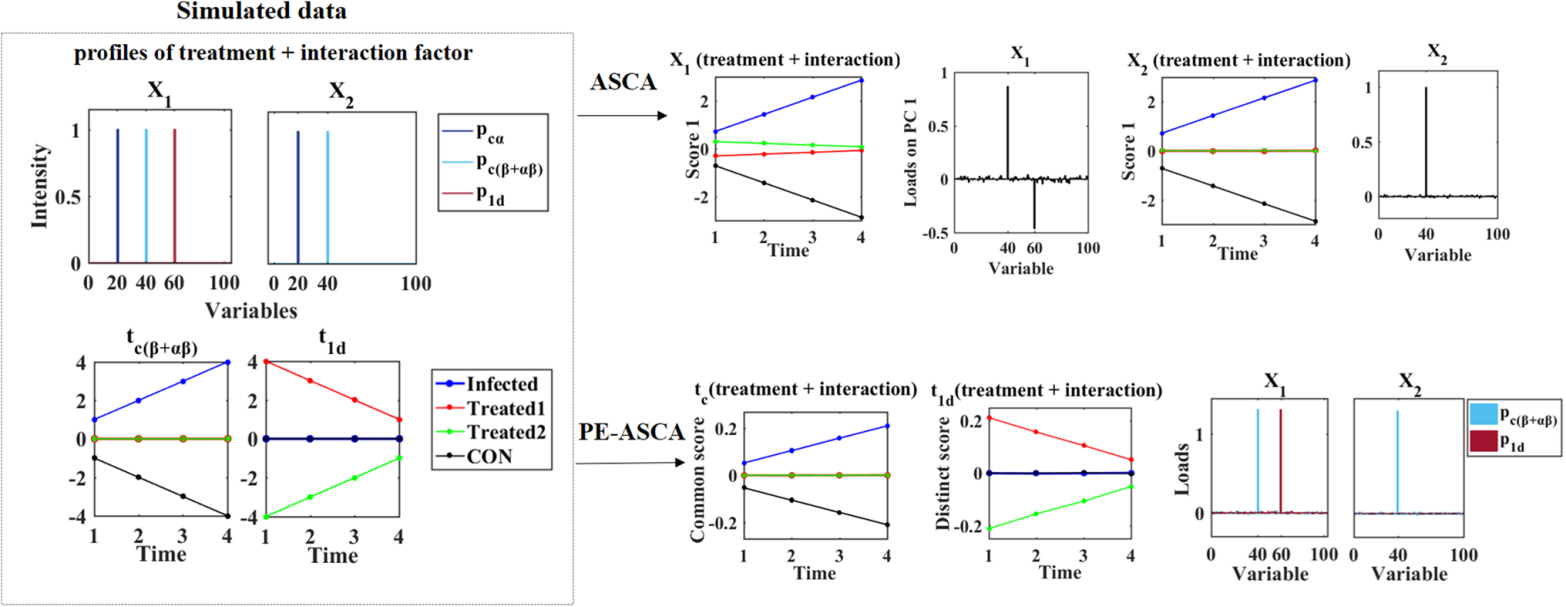
Simulated data for the case B and the scores over different time points and loadings obtained ASCA and PE-ASCA model

The time effect has four levels (i.e. four time points where the level increases with time). The treatment effect has four levels with level one related to infected piglets, level two represents the infected piglets treated with the first type of supplementation (Treated1), level three is the infected piglets treated with the second type of supplementation (Treated2), and level four is the uninfected control piglets (CON). Variable 20 is responsible for the common component of time effect, while variable 40 is responsible for the common component for the combination of treatment and treatment × time effect. The score matrices are simulated to be orthogonal to each other in each time point. Therefore, in the common score, the Treated1 and Treated2 groups are zero over all time points, while the infected and CON groups are zero over all time points in the distinct score of first data. One distinct component is also explaining the extra variation related to the combination of treatment and treatment × time in the first data in variable 60. In this case, the second data does not contain a distinct component. The common and distinct scores for simulating the combination of treatment and treatment × time effects as well as the simulated loadings can be seen in Fig. 3.

Case B is simulated to illustrate the advantage of PE-ASCA model in comparison to the ASCA model of single data analysis. As a result of the ASCA model, the variation of each data set is distributed based on the experimental design of the data. Thus, the variation of the time factor is separated from the combined treatment and treatment × time effect. Variable 20 is related to the time factor (not shown). As can be seen from the ASCA result of the first data (Fig. 3), both common and distinct effects are present in the first score of the mean values over time. It means that there is an increasing trend of treatment levels of infected and CON groups, while the level of the other two groups is decreasing over time. The corresponding loading plot shows the contribution of both variables 40 and 60. In the ASCA results of the second data, only an increasing trend in levels of infected and CON over time is present. Permutation test on effect matrices showed that the observed effect for time and combination of treatment and treatment × time are significant in both data sets (p value = 0.001 for 1000 iterations). On the other hand, the results of the PE-ASCA model show the ability of the model to correctly distinguish the common and distinct variation of *β* + *αβ* effect. More importantly, investigation of the loading plots shows that the variables are correctly disentangled for the common and distinct variation and no significant value can be seen for variable 60 in the common component.

### Experimental example and data acquisition

The PE-ASCA model is also implemented on a real NMR metabolomics data set obtained from different brains tissues of piglets in an intervention study of experimental bloodstream infection. The data was already presented in its original paper (Alinaghi et al. 2019). Here, a sub-set of data is re-analyzed with the PE-ASCA model.

**Fig. 4:**
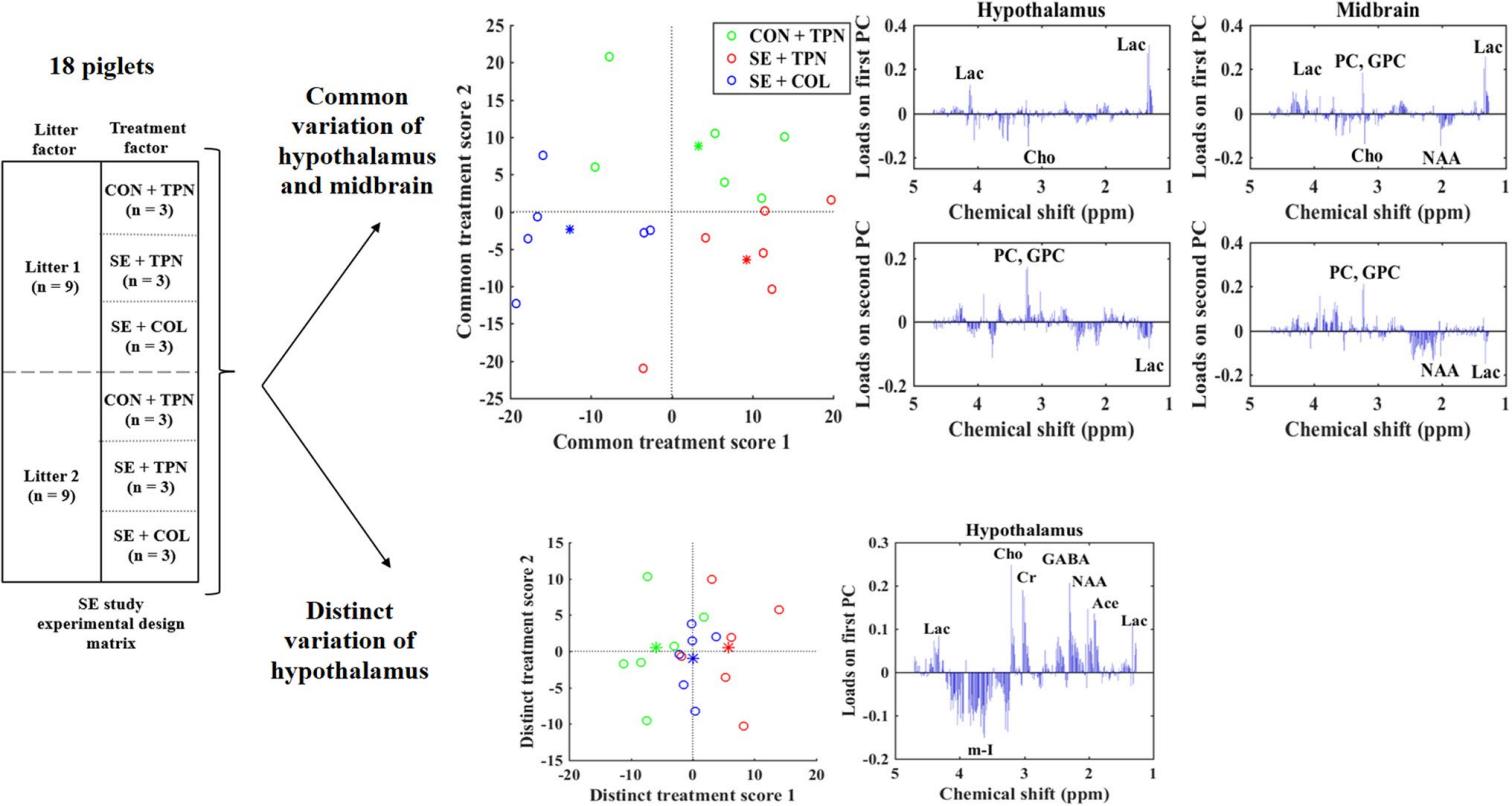
PE-ASCA result of the experimental NMR-metabolomics data, **a** schematic overview of the balanced experimental design of the SE study, **b** common score and loading plots of hypothalamus and midbrain, **c** distinct score and loading plots of hypothalamus. CON + TPN (green circles) represents the control group, SE + TPN (red circles) represents the infected group by *Staphylococcus epidermidis* and SE + COL (blue circles) represents the colostrum supplementation group. The stars represent the mean value of each treatment group. In the loading plots: *Ace* acetate, *GABA* γ-aminobutyric acid, *GPC* glycerophosphocholine, *Cho* choline, *Cr* creatine, *Lac* lactate, *m*-*I myo*-inositol, *NAA N*-acetylaspartate, *PC* phosphocholine (Color figure online)

In the experimental data, twenty-two piglets (Danish landrace × Large White × Duroc) were delivered at 90% gestation from two sows (defined as litter 1 and litter 2), while each delivered piglet were randomly allocated to one of the following three treatment groups: (i) a control group receiving intra-arterial saline and total parenteral nutrition (CON + TPN, *n* = 7), (ii) an infected group receiving *Staphylococcus epidermidis* (SE) inoculation and total parenteral nutrition (SE + TPN, *n* = 5) or (iii) a colostrum supplementation group receiving the SE inoculation and bovine colostrum and supplementary parenteral nutrition (SE + COL, *n* = 10). Therefore, each piglet is attributed to a specific level of litter and treatment. A schematic overview of the experimental design is depicted in Fig. 4a. The brain regions of hypothalamus and midbrain were analyzed by HR-MAS NMR spectroscopy. A Bruker Avance III 600 MHz NMR spectrometer (Bruker BioSpin Gmbh, Rheinstetten, Germany) equipped with a 5-mm HR-MAS probe was used for recording the ^1^H NMR spectrum at 281 K with a spinning speed of 5000 Hz. Detailed descriptions of nutritional compositions of the intervention diets and NMR data acquisition are available elsewhere (Alinaghi et al. 2019; Brunse et al. 2018; Shen et al. 2015). In order to have a balanced design in the data, four samples from SE + COL group and one sample from CON + TPN group were removed from the data sets. The samples were chosen to be removed if they were outliers, otherwise they were randomly selected. Moreover, one sample was reconstructed by adding the normally distributed noise to the mean response of the SE + TPN samples in the first litter. The final NMR data set of hypothalamus and midbrain had the size of 18 × 330 containing the three samples in every treatment group of each litter and were used for the integrative analysis with PE-ASCA model.

For pre-processing of the data prior to analysis, the data were referenced to the trimethylsilyl-[2,2,3,3-^2^H_4_]-1-propionate (TSP) signal at 0.0 ppm and phase- and baseline-corrected. The spectral data was aligned through the local shifting of customized intervals by interval-correlation-shifting algorithm (icoshift) (Savorani et al. 2010). The data were normalized to the total sum area under the peak and subdivided into 0.01 ppm bins. The pareto-scaled aliphatic part (1.3 – 4.5 ppm) of the data, excluding the spectral regions containing ethanol and propylene glycol resonances (Alinaghi et al. 2019), was used for analysis.

A permutation test showed that the observed common litter and treatment effect are significant (p value = 0.003 for litter effect and p value = 0.04 for treatment effect with 1000 iterations). The PE-ASCA results show that two components can be disentangled for the common treatment effect (Fig. 4b). The score plot shows that the variation related to the separation of the SE + TPN and CON + TPN groups from the other group can be explained by the first common PC, while the separation of the CON + TPN group from the other two groups can be explained by the second common PC. Inspection of the loading plots, which contains the spectral data that can be translated into specific metabolites, reveals that the metabolite lactate can similarly explain the separation of the SE + TPN group in data sets of both compartments, while lactate is increased in intensity in the SE + TPN group. On the other hand, the alternation of the choline-containing metabolites, which is displayed in the loading plots, is of importance while the metabolites glycerophosphocholine (GPC) and phosphocholine (PC) show a higher level in CON + TPN group in midbrain compartment.

For the midbrain data, the distinct treatment effect is not significant. It shows that there is no further variation in the midbrain data to explain the treatment effect. However, the distinct treatment effect in the hypothalamus data shows a p value = 0.06. The distinct score plot, explaining the treatment variation in hypothalamus, is shown in Fig. 4c. Only the first distinct score illustrates further variation explaining the separation of two groups of CON + TPN and SE + TPN from each other. This separation is explaining a higher level of choline in the infected group as well as some other metabolites such as γ-aminobutyric acid, N-acetylaspartate and acetate.

## Discussion

Ignoring the experimental design information can hamper the interpretation of the results in PCA. Therefore, ASCA have been developed to implement the available information about the experimental design for a better modeling and successively better understanding of the biological system. The same approach can also be considered for analysis of the fused data sets, which could be more complicated than analysis of a single data. Implementing the information about experimental design can lead to better understanding of the common and distinct phenomena in integrative analysis of multiple data sets.

The simulated data in case A illustrated the advantages of the proposed model in comparison with data fusion without consideration of the experimental design. The results represent the capability of the PE-ASCA in correctly estimating the all designed factors in this high signal to noise ratio scenario where SCA and PE-SCA could not recover some factors. Here, SCA and P-ESCA are investigated for the comparison; however, the benefit of PE-ASCA can be extended to other fusion methods such as JIVE and SLIDE which do not use the available experimental design information. Moreover, analysis of the individual differences could also be informative as it can reveal the common and distinct variation which is not part of the experimental design of the data sets.

The need for data fusion methods and their advantages in providing a deeper understanding of the biological systems are vastly discussed in the literature (Smilde et al. 2017; Smilde et al. 2005b; Song et al. 2019).The benefit of an integrative analysis of multiple data sets with the similar underlying experimental design is presented here. As demonstrated with the analysis of the simulated data in case B, the common and distinct sources of information obtained from the two (or multiple) sets can be affected by each of the design factors or even their interaction. By taking the design effect into consideration when analyzing fused data sets, complementary knowledge about the underlying experimental variation in the biological system can be obtained.

Furthermore, the analysis of real experimental data illustrated the usefulness of the proposed model. This was done on brain metabolome data from an intervention study with pigs, and the results indicate that lactate levels are higher in the hypothalamus and midbrain sections of the infected piglets. This is consistent with the conversion of glucose to lactate, which can often be observed during sepsis conditions as a result of the glycolytic shift under anaerobic conditions, and it was also observed in the analysis of cerebrospinal fluid and plasma samples from piglets in this study (Alinaghi et al. 2019). However, a glucose signal could not be detected in the HR-MAS data of brain samples. Regarding the choline-containing metabolites (Corbin and Zeisel 2012), the infected piglets exhibited a higher level of PC and GPC, which may be associated to the alternation of membrane choline phospholipid metabolism, and possibly the severity of the cerebral damage. Choline is a metabolite present in the cell and mitochondrial membrane and is essential for development of the brain and lipid metabolism (Khovidhunkit et al. 2004).

## Conclusions

In this study, an integrative analysis of multiple designed data sets is investigated by incorporating the experimental design information in a data fusion model. In this way, the proposed PE-ASCA model can reveal the common and distinct variation associated with various sources of experimental factors (such as time, treatment and litter) in the data sets. Therefore, this method can provide easier interpretation of induced metabolite alternations in fusion of multiple data sets from different biological compartments or analytical platforms.

## Electronic supplementary material

Below is the link to the electronic supplementary material.

Supplementary material 1 (DOCX 648 kb)
