# Supplementary material for: Common and distinct variation in data fusion of designed experimental data
Source: Metabolomics. 2019 Dec 3;16(1):2. doi: 10.1007/s11306-019-1622-2 (PMC6890597; doi:10.1007/s11306-019-1622-2)
Supplement: Supplementary file 1 — Supplementary material 1 (DOCX 648 kb) [file 11306_2019_1622_MOESM1_ESM.docx]

**Supplementary material**

**Title:** Common and distinct variation in data fusion of designed experimental data

**Authors:** Masoumeh Alinaghi^1^, Hanne Christine Bertram^1^, Anders Brunse^2^, Age K. Smilde^3^, Johan A. Westerhuis^3,*^

^1^ Department of Food Science, Aarhus University, Aarslev, Denmark

^2^ Comparative Pediatrics and Nutrition, Department of Veterinary and Animal Sciences, Faculty of Health and Medical Sciences, University of Copenhagen, Denmark

^3^ Biosystems Data Analysis, Swammerdam Institute for Life Sciences, University of Amsterdam, Amsterdam, The Netherlands

* Correspondence [j.a.westerhuis@uva.nl](mailto:j.a.westerhuis@uva.nl)

**Two different approaches for PE-ASCA model**

Considering the concept of decomposing multiple data sets into common and distinct variation for each experimental factor, either incorporating the ASCA model on the P-ESCA decomposed matrices (approach I) or fusion of the ASCA-decomposed effect matrices (approach II) will theoretically lead to the same results. Eq. (S1) and (S2) prove that these two approaches can result in the mathematically same results in ideal data sets without noise. Suppose that the data set $\mathbf{X}_{1}$and $\mathbf{X}_{2}$are mean centered, then decomposition of the data sets is according to Eq. (S1). For simplicity, it is considered that there is no interaction between factors α and β.

| $\mathbf{X}_{1}\boldsymbol{=}\mathbf{T}_{c\alpha}\mathbf{P}_{1c\alpha}^{T}+\mathbf{T}_{c\beta}\mathbf{P}_{1c\beta}^{T}+\mathbf{T}_{1d\alpha}\mathbf{P}_{1d\alpha}^{T}+\mathbf{T}_{1d\beta}\mathbf{P}_{1d\beta}^{T}$  $\mathbf{X}_{2}\boldsymbol{=}\mathbf{T}_{c\alpha}\mathbf{P}_{2c\alpha}^{T}+\mathbf{T}_{c\beta}\mathbf{P}_{2c\beta}^{T}+\mathbf{T}_{2d\alpha}\mathbf{P}_{2d\alpha}^{T}+\mathbf{T}_{2d\beta}\mathbf{P}_{2d\beta}^{T}$ | (S1) |
| --- | --- |

Where theoretically all scores $\mathbf{T}_{c\alpha}$, $\mathbf{T}_{c\beta}$, $\mathbf{T}_{1d\alpha}$, $\mathbf{T}_{1d\beta}$, $\mathbf{T}_{2d\alpha}$ and $\mathbf{T}_{2d\beta}$are orthogonal to each other, $\mathbf{T}^{T}\mathbf{T}=\mathbf{I}$. However, due to the applied penalty function in the P-ESCA model, this requirement will not be obtained in practice.

In approach I, the data sets $\mathbf{X}_{1}$and $\mathbf{X}_{2}$ are decomposed into the common and distinct variation according to the P-ESCA model (Eq. (S2)).

| $\mathbf{X}_{1}\boldsymbol{=}\mathbf{X}_{1c}\boldsymbol{+}\mathbf{X}_{1d}$  $\mathbf{X}_{\boldsymbol{2}}\boldsymbol{=}\mathbf{X}_{2c}\boldsymbol{+}\mathbf{X}_{2d}$ | (S2) |
| --- | --- |

ASCA decomposes the matrices $\mathbf{X}_{1c}$, $\mathbf{X}_{1d}$, $\mathbf{X}_{2c}$and $\mathbf{X}_{2d}$in Eq. (S2) into the sub-matrices describing *α* and *β* variation (Eq. (S3)).

| $\mathbf{X}_{1c}\boldsymbol{=}{\mathbf{X}_{1c\alpha}\boldsymbol{+}\mathbf{X}_{1c\beta}\mathbf{=T}}_{c\alpha}\mathbf{P}_{1c\alpha}^{T}+\mathbf{T}_{c\beta}\mathbf{P}_{1c\beta}^{T}$  ${\mathbf{X}_{1d}\mathbf{=}\mathbf{X}_{1d\alpha}\boldsymbol{+}\mathbf{X}_{1d\beta}\mathbf{=T}}_{1d\alpha}\mathbf{P}_{1d\alpha}^{T}+\mathbf{T}_{1d\beta}\mathbf{P}_{1d\beta}^{T}$  $\mathbf{X}_{2c}\boldsymbol{=}{\mathbf{X}_{2c\alpha}\boldsymbol{+}\mathbf{X}_{2c\beta}\mathbf{=T}}_{c\alpha}\mathbf{P}_{2c\alpha}^{T}+\mathbf{T}_{c\beta}\mathbf{P}_{2c\beta}^{T}$  ${\mathbf{X}_{2d}\mathbf{=}\mathbf{X}_{2d\alpha}\boldsymbol{+}\mathbf{X}_{2d\beta}\mathbf{=T}}_{2d\alpha}\mathbf{P}_{2d\alpha}^{T}+\mathbf{T}_{2d\beta}\mathbf{P}_{2d\beta}^{T}$ | (S3) |
| --- | --- |

Where, $\mathbf{T}_{c\alpha}^{T} \mathbf{T}_{c\beta}=0$, $\mathbf{T}_{1d\alpha}^{T} \mathbf{T}_{1d\beta}=0$ and $\mathbf{T}_{2d\alpha}^{T} \mathbf{T}_{2d\beta}=0$.

In approach II, the data sets $\mathbf{X}_{1}$and $\mathbf{X}_{2}$ are decomposed into the sub-matrices describing *α* and *β* variation (Eq. (S4)).

| $\mathbf{X}_{1}\boldsymbol{=}\mathbf{X}_{1\alpha}\boldsymbol{+}\mathbf{X}_{1\beta}$  $\mathbf{X}_{\boldsymbol{2}}\boldsymbol{=}\mathbf{X}_{2\alpha}\boldsymbol{+}\mathbf{X}_{2\beta}$ | (S4) |
| --- | --- |

P-ESCA disentangles the common and distinct components by integrative analysis of $\mathbf{X}_{1\alpha}$ and $\mathbf{X}_{2\alpha}$as well as integrative analysis of $\mathbf{X}_{1\beta}$and $\mathbf{X}_{2\beta}$ (Eq. (S5)). .

| $\mathbf{X}_{1\alpha}\boldsymbol{=}{\mathbf{X}_{1c\alpha}\boldsymbol{+}\mathbf{X}_{1d\alpha}\mathbf{=T}}_{c\alpha}\mathbf{P}_{1c\alpha}^{T}+ \mathbf{T}_{1d\alpha}\mathbf{P}_{1d\alpha}^{T}$  $\mathbf{X}_{1\beta}\boldsymbol{=}{\mathbf{X}_{1c\beta}\boldsymbol{+}\mathbf{X}_{1d\beta}\mathbf{=T}}_{c\beta}\mathbf{P}_{1c\beta}^{T}+\mathbf{T}_{1d\beta}\mathbf{P}_{1d\beta}^{T}$  $\mathbf{X}_{2\alpha}\boldsymbol{=}{\mathbf{X}_{2c\alpha}\boldsymbol{+}\mathbf{X}_{2d\alpha}\mathbf{=T}}_{c\alpha}\mathbf{P}_{2c\alpha}^{T}+\mathbf{T}_{2d\alpha}\mathbf{P}_{2d\alpha}^{T}$  ${\mathbf{X}_{2\beta}\boldsymbol{=}\mathbf{X}_{2c\beta}\boldsymbol{+}\mathbf{X}_{2d\beta}\mathbf{=T}}_{c\beta}\mathbf{P}_{2c\beta}^{T}+\mathbf{T}_{2d\beta}\mathbf{P}_{2d\beta}^{T}$ | (S5) |
| --- | --- |

Where, $\mathbf{T}_{c\alpha}^{T} \mathbf{T}_{1d\alpha}=0$, $\mathbf{T}_{c\beta}^{T} \mathbf{T}_{1d\beta}=0$, $\mathbf{T}_{c\alpha}^{T} \mathbf{T}_{2d\alpha}=0$ and $\mathbf{T}_{c\beta}^{T} \mathbf{T}_{2d\beta}=0$.

Comparison of these two approaches can be seen in Fig. S1, which illustrates the same results for ASCA model on the P-ESCA decomposed matrices and P-ESCA model on the multiple ASCA-decomposed effect matrices. This figure illustrates the column space where common variation has the same **T** scores; however, their **P** matrices are not identical. This could be different in analysis of real data with PE-ASCA model, due to penalty impose and presence of noise.

| 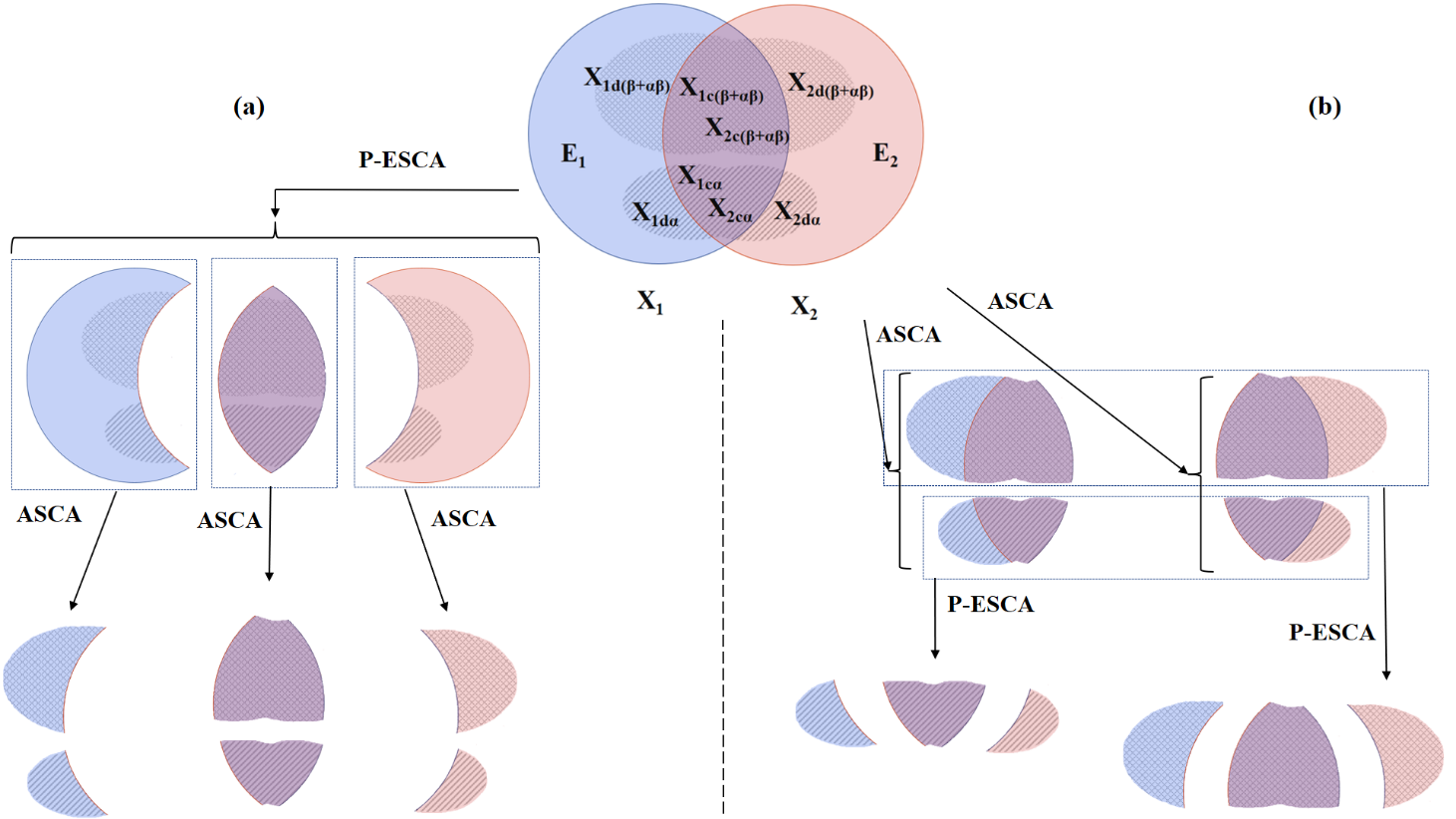 |
| --- |
| Fig. S1. Schematic comparison of (a) approach I and (b) approach II for disentangling the common and distinct parts of multiple designed experimental data sets. |
